# Characterization and modulation of drug resistance of human paediatric rhabdomyosarcoma cell lines

**DOI:** 10.1054/bjoc.2000.1273

**Published:** 2000-07-03

**Authors:** H A Cocker, C R Pinkerton, L R Kelland

**Affiliations:** CRC Centre for Cancer Therapeutics, The Institute of Cancer Research, Cotswold Road, Sutton, Surrey, SM2 5NG, UK; Section of Paediatrics, The Institute of Cancer Research/Royal Marsden NHS Trust, Cotswold Road, Sutton, Surrey, SM2 5NG, UK

**Keywords:** rhabdomyosarcoma, MDR-1, resistance, modulation

## Abstract

The role of multidrug resistance (MDR) and *p53* functional status in the treatment of paediatric rhabdomyosarcoma is unclear. We have characterized a panel of seven human rhabdomyosarcoma cell lines for MDR and *p53* phenotype. None of the cell lines had P-glycoprotein (P-gp) or multidrug resistance-related protein (MRP) detectable by Western blotting, whereas immunohistochemistry suggested that very low levels of MDR proteins may be present in some of the lines. RT-PCR studies indicated that *mdr-1*, *mrp-1* and *lrp* mRNA was present in 5/7, 7/7 and 5/7 lines respectively. The function of *p53* is compromised in six of the lines, either through mutation of the *p53* gene or by overexpression of *mdm-2*. The sensitivity of many of the cell lines to vincristine could be modulated above 2-fold and as high as 16-fold using two modulating agents, PSC833 and VX710 (with VX710 being a significantly more potent modulator of the rhabdomyosarcoma lines). PSC833 also increased vincristine accumulation in all of the lines from 1.2- to 2.2-fold. These results suggest that some of these cell lines have low levels of multidrug resistance. The level of MDR proteins is very low and therefore difficult to detect, but may be sufficient to confer low-level, but clinically relevant, resistance to some cytotoxic agents, especially vincristine. These cell lines will therefore provide a suitable model to test new strategies in treatment and for further understanding relationships between protein expression and drug resistance. © 2000 Cancer Research Campaign


*British Journal of Cancer* (2000) **83**(3), 338–345
© 2000 Cancer Research Campaign
doi: 10.1054/ bjoc.2000.1273, available online at http://www.idealibrary.com on IDEAL®

### Characterization and modulation of drug resistance of human paediatric rhabdomyosarcoma cell lines

**HA Cocker[1], CR Pinkerton[2] and LR Kelland[1]**

[1]CRC Centre for Cancer Therapeutics, The Institute of Cancer Research, Cotswold Road, Sutton, Surrey, SM2 5NG, UK; [2]Section of Paediatrics, The Institute of Cancer Research/Royal Marsden NHS Trust, Cotswold Road, Sutton, Surrey, SM2 5NG, UK

**Summary** The role of multidrug resistance (MDR) and *p53* functional status in the treatment of paediatric rhabdomyosarcoma is unclear. We have characterized a panel of seven human rhabdomyosarcoma cell lines for MDR and *p53* phenotype. None of the cell lines had P-glycoprotein (P-gp) or multidrug resistance-related protein (MRP) detectable by Western blotting, whereas immunohistochemistry suggested that very low levels of MDR proteins may be present in some of the lines. RT-PCR studies indicated that *mdr-1*, *mrp-1* and *lrp* mRNA was present in 5/7, 7/7 and 5/7 lines respectively. The function of *p53* is compromised in six of the lines, either through mutation of the *p53* gene or by overexpression of *mdm-2*. The sensitivity of many of the cell lines to vincristine could be modulated above 2-fold and as high as 16-fold using two modulating agents, PSC833 and VX710 (with VX710 being a significantly more potent modulator of the rhabdomyosarcoma lines). PSC833 also increased vincristine accumulation in all of the lines from 1.2- to 2.2-fold. These results suggest that some of these cell lines have low levels of multidrug resistance. The level of MDR proteins is very low and therefore difficult to detect, but may be sufficient to confer low-level, but clinically relevant, resistance to some cytotoxic agents, especially vincristine. These cell lines will therefore provide a suitable model to test new strategies in treatment and for further understanding relationships between protein expression and drug resistance. © 2000 Cancer Research Campaign

**Keywords**: rhabdomyosarcoma; MDR-1; resistance; modulation

Rhabdomyosarcoma is a highly malignant soft-tissue sarcoma that occurs primarily in childhood. It accounts for 6–7% of all cancers in children and over half of all paediatric soft-tissue sarcomas (Enzinger and Weiss, 1995). It is classified according to histology and the two major classifications in paediatric cases are alveolar and embryonal (Enzinger and Weiss, 1983). The alveolar form usually has a specific chromosomal t(2;13)(q35;q14) translocation which gives rise to a novel fusion protein (Douglass et al, 1987). The presence of this translocation correlates with a worse prognosis and treatment is therefore adjusted accordingly. Drugs commonly used for treatment include vinca alkaloids, anthracyclines, etoposide and cyclophosphamide.

The long-term survival rate for paediatric rhabdomyosarcoma is now approximately 70%, compared to 20% in 1970 (Pappo et al, 1995). This recent improvement is largely due to the use of more effective multi-agent chemotherapy. Despite this, relapse due to drug resistance remains a major obstacle to survival. It has become apparent in recent years that multidrug resistance (MDR) may be one of the main reasons for this. In rhabdomyosarcoma, an association between the presence of P-glycoprotein (P-gp, encoded by the gene *mdr-1*) and adverse prognosis has been documented (Chan et al, 1990) but more recent studies have failed to confirm this (Kuttesch et al, 1996). More recently, other mechanisms such as multidrug resistance-related protein (MRP) (Cole et al, 1992) and lung cancer resistance protein (LRP) (Scheper et al, 1993) have also been implicated in the MDR phenotype, although not specifically in rhabdomyosarcoma. LRP sequesters drugs thereby reducing their availability in the cell. P-gp and MRP efflux a variety of drugs used to treat cancer, thereby reducing the availability of drug at the target and reducing the effectiveness of treatment.

Ways of reversing or modulating multidrug resistance have been researched extensively in recent years and several compounds have been found which affect the MDR mechanisms (reviewed by Ford, 1996). These include the immunosuppressive cyclosporin A and the calcium channel-blocker verapamil. The effectiveness of these agents in rhabdomyosarcoma in vitro has been investigated (Cowie et al, 1998). However, the use of these agents as MDR modulators in vivo is limited by toxicity due to side-effects such as nephrotoxicity for cyclosporin A and cardiac toxicity for verapamil.

Currently one of the most promising MDR modulators is the non-immunosuppressive cyclosporin A analogue, PSC833, which is specific for P-gp (Naito and Tsuruo, 1997). This is currently undergoing phase III clinical trials in relapsed acute myeloid leukaemia and in relapsed or refractory multiple myeloma. The compound has also undergone several phase I and II trials in various types of cancer. Another compound is the novel modulator VX710 (Germann et al, 1997*a*), a non-macrocyclic pipecolinate derivative which binds the FK506 receptor protein. It inhibits the efflux activity of P-glycoprotein and stimulates vanadate sensitive P-glycoprotein ATPase activity, suggesting a direct high-affinity interaction between VX710 and P-glycoprotein (Germann et al, 1997*a*). VX710 has also been shown to modulate MRP-mediated multidrug resistance, possibly by direct interaction (Germann et al,

*Received 22 September 1999*
*Revised 13 January 2000*
*Accepted 24 March 2000*

*Correspondence to:* LR Kelland

**Table 1** Rhabdomyosarcoma cell line panel

| Cell line | Histological subtype | Prior patient treatment | Reference |
|---|---|---|---|
| Rh18 | Mixed | Untreated | Houghton et al, 1982 |
| Rh30 | Alveolar | Untreated | Douglass et al, 1987 |
| Rh36 | Embryonal | Multiagent chemotherapy | Keleti et al, 1996 |
| SCMC | Alveolar | Multiagent chemotherapy[a] | Hayashi et al, 1990 |
| HX170 | Embryonal | Multiagent chemotherapy[b] and radiotherapy | Kelland et al, 1989 |
| RMS | Alveolar | Multiagent chemotherapy[c] | Garvin et al, 1986 |
| RD | Embryonal | Cyclophosphamide and radiotherapy | McAllister et al, 1969 |

[a]Vincristine, cyclophosphamide, doxorubicin and dactinomycin; [b]vincristine, cyclophosphamide and doxorubicin; [c]vincristine, cyclophosphamide and actinomycin D

1997*b*). This agent is currently in phase II clinical trials in breast cancer, ovarian cancer, soft-tissue sarcoma, liver cancer and prostate cancer.

MDM2 protein binds and inactivates the tumour suppressor protein p53 (Lane, 1992) and has recently been implicated in the development of drug resistance (Kondo et al, 1996). It is known that *mdm*2 is often overexpressed in sarcomas and in particular in rhabdomyosarcoma (Keleti et al, 1996). The study also showed that when *mdm2* expression was normal, the tumour suppressor gene, *p53*, was often mutant. Separate studies have shown that *p53* mutations are often present in childhood rhabdomyosarcomas, particularly at relapse (Felix et al, 1992; Wurl et al 1996).

In the present study a panel of seven paediatric rhabdomyosarcoma cell lines have been characterized with regard to *p53*, *p21* and *mdm*2 in addition to drug resistance phenotype and sensitivity to the commonly used anti-cancer drugs, cisplatin, doxorubicin, etoposide and vincristine. The ability of two different drug resistance modulators to improve sensitivity has also been investigated.

### MATERIALS AND METHODS

### Cell lines

The panel of rhabdomyosarcoma cell lines is summarized in Table 1. Rh18, Rh30 and Rh36 were gifts from Dr PJ Houghton (St Judes Children's Research Hospital). SCMC was a gift from T Sawada (Kyoto Prefectural University of Medicine, Japan). RMS and RD were obtained from the American Tissue Culture Collection (Rockville, Maryland, USA). All the rhabdomyosarcoma lines used are negative for EWS-FLI-1 (Dr A Gordon, personal communication). The lines Rh18, Rh30 and RMS have the PAX3-FKHR translocation associated with t(2;13)(q35;q14) and the line SCMC has a t(9;13) translocation, which does not affect the FKHR gene, in addition to a cryptic der(13)t(2;13)(q35;q14) translocation (Dr A Gordon, personal communication).

The human ovarian carcinoma cell line CH1 and its multidrug resistant variant CHIDoxR were used as negative and positive controls for P-glycoprotein expression and function (Sharp et al, 1994). The human non-small cell lung carcinoma cell line CORL23 and its multidrug resistant variant CORL23/R were used as negative and positive controls for MRP expression (Barrand et al, 1994). The non-small cell lung cancer cell line 2R120 and the breast cancer cell line MCF-7 were used as positive and negative controls respectively for LRP expression (Scheper et al, 1993). The ovarian cell line A2780 was used as a wild-type *p53* control (Pestell et al, 1998).

All cell lines were maintained in RPMI 1640 medium (Sigma, Poole, Hants, UK) supplemented with 10% foetal bovine serum (Life Technologies, Scotland, UK) and 2 mM L-glutamine (Sigma). Cells were grown as attached monolayers and were incubated at 37°C in a humidified atmosphere with 5% $CO_2$. All cell lines were routinely screened for *Mycoplasma* by PCR assay (Stratagene, Cambridge, UK).

### Drugs and chemicals

Vincristine (David Bull Laboratories) and etoposide (Bristol Myers Squibb Pharmaceuticals) stock solutions were obtained as pharmacy preparations at concentrations of 1.083 mM and 34 mM respectively. Doxorubicin (Sigma) was dissolved in water to give a stock solution of 1 mM. Cisplatin (Johnson Matthey Technology Centre, Reading, UK) was dissolved in 0.9% saline to give a stock solution of 1 mM. VX710 (Vertex Pharmaceuticals Inc, Cambridge, MA, USA) was dissolved in saline to give a 50 mM stock solution. Finally, PSC833 (Sandoz Pharmaceuticals, Camberley, UK) was dissolved in absolute ethanol to give a 1 mM stock. All of these drugs were stored at –20°C with the exception of vincristine which was stored at 4°C. Immediately prior to use the drugs were diluted using RPMI 1640 medium prepared as for cell culture, with the exception of vincristine which was diluted in RPMI 1640 medium without any supplements.

MTT (3-(4,5-dimethylthiazol-2-yl)-2,5-diphenyltetrazolium bromide) (Sigma) was dissolved in PBS to produce a stock solution of 5 mg $ml^{-1}$ which was stored at 4°C.

### Immunoblotting

Exponentially growing cells were harvested by trypsinization, washed in PBS then incubated in 200–300 µl of lysis buffer (pH 7.5, 150 mM NaCl, 50 mM Tris-HCl, 1 mM phenylmethylsulphonyl fluoride (PMSF), 2 µg $ml^{-1}$ aprotinin, 2 µg $ml^{-1}$ leupeptin, 1 mM sodium orthovanadate, 1% NP40 and 0.2% sodium dodecyl sulphate (SDS) at 4°C for 1 h. Cells were then centrifuged at 7000 ***g*** (MSE Microcentrifuge) at 4°C for 15 min. The protein-containing supernatant was collected and stored at –70°C.

When immunoblotting for P-glycoprotein a membrane protein preparation from the cells was used. Cells were harvested by trypsinization, washed with PBS then lysed in 1 mM Tris (pH 7.4)

© 2000 Cancer Research Campaign containing 100 μg $ml^{-1}$ PMSF at 4°C for 1 h. Nuclei and unbroken cells were removed by centrifugation (450 ***g***, 10 min, 4°C). The resulting supernatant was centrifuged (60 000 ***g***, 1 h, 4°C) to pellet the cell membranes. The membrane proteins were resuspended in lysis buffer and stored at –70°C.

The protein content of the samples was determined using a BCA assay kit (Pierce, Rockford, IL, USA). Cell lysates were then diluted 1:1 in 2× Laemmli buffer (62.5 mM Tris-HCl pH 6.8, 20% (v/v) glycerol, 10% (v/v) β-mercaptoethanol, 5% (w/v) SDS, 0.005% (w/v) bromophenol blue) and denatured for 3 min at 95°C. Proteins were resolved by electrophoresis down an 8–16% SDS-PAGE gradient gel as previously described (Sharp et al, 1995). When immunoblotting for P-gp or MRP, a 6% gel was used, to ensure better separation of the protein of interest. Equal amounts of protein from each sample were loaded into each well, typically 60 μg.

Proteins were then electroblotted onto a nitrocellulose filter (Millipore) (Towbin et al, 1979) in a transfer buffer containing 10% methanol at 300 mA for 2 h at 4°C. Even loading and transfer of proteins was assessed by staining with Ponceau S. The nitrocellulose filter was then blocked overnight in PBS (pH 7.6) containing 0.5% casein. Target proteins were detected using a primary antibody and an appropriate horseradish peroxidase-labelled secondary antibody followed by enhanced chemiluminescence reagents (Amersham). Exposure of the nitrocellulose filter to film (Hyperfilm-ECL, Amersham) was used to visualize protein bands. The antibodies used were C219 (Centocor, USA) to detect P-gp, MRPm6 (Monosan, Netherlands) to detect MRP, IF-2 (Oncogene, Cambridge, MA, USA) to detect MDM2, C19 (Santa Cruz Biotechnology, Santa Cruz, CA, USA) to detect p21, DO-1 (Santa Cruz Biotechnology) to detect p53 and B-1-5-2 (Sigma) to detect α-tubulin.

### Immunohistochemistry

Cytospin preparations of approximately 50 000 cells were fixed in acetone (for MRP and LRP) or 4% paraformaldehyde (P-gp) for 5 min, air-dried and pre-incubated in 10% rabbit serum in TBS. This was followed by incubation with primary antibody for 1 h. The antibodies used were MRK 16 (TCS Biologicals, Botolph Claydon) diluted 1:50 to detect P-gp, MRPm6 diluted 1:100 to detect MRP and LRP 56 (TCS Biologicals) diluted 1:20 to detect LRP. All antibodies were titrated to ensure maximum sensitivity. Incubation with the primary antibody was followed by incubation for 30 min with rabbit anti-mouse antibody (Dako, Glastrup, Denmark) diluted 1:50. A standard APAAP technique (Dako) was used for colourimetric development with levamisole (Sigma) to block activity of endogenous alkaline phosphatase. All incubations were carried out at room temperature. Slides were counterstained with Mayer's haematoxylin (Sigma) for 2 min. Cells were graded as negative (–) when no staining was apparent, positive (+) when staining was clear and unclear (–/+) when staining was difficult to detect. In all cases, appropriately matched isotype controls and no antibody controls were used.

### Reverse transcriptase-polymerase chain reaction (RT-PCR)

All sequences of PCR primers were as previously reported. RT-PCR analysis used the following primer sets: *mdr-1*, 157 bp product (Bordow et al, 1994), *mrp-1*, 140 bp product (Bordow et al, 1994) and *lrp*, 405 bp product (Stein et al, 1997). All primer sets spanned an intron to control against amplification of genomic DNA sequences. All primers were purchased from Oswel DNA Service Lab (Southampton, UK).

Total RNA (1 μg) was reversed transcribed in a 20 μl volume using Superscript (GIBCO BRL, Scotland, UK) and random hexonucleotides (Pharmacia), according to the manufacturer's instructions. PCR was performed in a final volume of 25 μl containing 0.5 μM of each primer, 0.2 mM dNTPs (Pharmacia), 2.5 U $\mu l^{-1}$ *Taq* DNA Polymerase (GIBCO BRL), *Taq* buffer (GIBCO BRL) and 1.5–2 mM $MgCl_2$ (GIBCO BRL). An initial denaturation for 4 min at 94°C was followed by 35 cycles of 30 s at 94°C, 60 s at 55°C and 60 s at 72°C in an Omnigene thermal cycler (Hybaid, UK). PCR products were visualized by electrophoresis using a 1.5% agarose gel and ethidium bromide staining.

### *p53* functional analysis

To provide some indication of the p53 functional status, induction of P53, P21 and MDM2 was measured following irradiation (O'Connor et al, 1997). Exponentially growing cells were exposed to 5 Gy irradiation delivered using a $^{60}Co$ source with a source-to-flask distance of 40 cm and a dose rate of 1.36 Gy $min^{-1}$. Cells were incubated for a further 4 h then harvested for protein extraction. The protein extracts were immunoblotted as above then probed for MDM2, P53 and P21. The proteins from irradiated cells were compared with protein from untreated cells to monitor changes in these proteins. The samples were also probed for α-tubulin to ensure even loading.

### Growth inhibition assay

The MTT (3-(4,5-dimethylthiazol-2-yl)-2,5-diphenyltetrazolium bromide) assay (Mosmann, 1983) was used to measure growth inhibition. Cells were added to each well of a flat bottom 96-well microtitre plate (Falcon, Becton Dickinson, Cowley, UK) at concentrations of $2 \times 10^3$–$1 \times 10^4$ cells per well. The seeding density was optimized to allow at least three cell doublings during the assay. The cytotoxic agents doxorubicin, vincristine, cisplatin and etoposide were added 24 h later. The cells were then incubated for a further 5 days (7 days for Rh18) after which the number of viable cells was determined using the MTT assay. Each experimental point was set up in quadruplicate wells and repeated on three separate occasions. The number of viable cells surviving drug exposure was expressed as a percentage of the number of viable cells in the absence of drug. This percentage was then plotted against drug concentration and from this the $IC_{50}$ (cytotoxic dose at which half the cells survive) was obtained.

The method was slightly modified in order to test the effect of the modulators PSC833 and VX710 on the chemosensitivity of the cell lines. The effects of the modulators alone were tested using an MTT assay similar to that outlined above. From this a concentration of modulator was chosen which killed no more than 10% of the cells in each cell line. This concentration was 2 μM for both modulators. The MTT assays were then carried out as above, except that after 24 h, modulator was added to each well before addition of the drug. The assay was then continued as normal. On each plate controls were included and any cytotoxic effects of the modulators were accounted for in the calculation of $IC_{50}$. The ratio of $IC_{50}$ with and without modulator treatment gives the sensitization ratio (SR) which was used to compare modulation between cell lines.

© 2000 Cancer Research Campaign

These experiments were carried out using the panel of seven rhabdomyosarcoma cell lines and also using CH1 and CH1DoxR as negative and positive controls for modulation.

### Vincristine accumulation assay

The accumulation of [$^3$H]-vincristine ([$^3$H]-VCR) was measured in exponentially growing cells. Cells were exposed to medium containing 10 nM or 25 nM [$^3$H]-VCR (Amersham, specific activity 6.2 Ci mmol$^{-1}$, 0.25 µCi µl$^{-1}$) and incubated at 37°C for 2 h. To test the effects of modulators on [$^3$H]-VCR accumulation, cells were incubated with 2 µM modulator 1 h prior to, and during, [$^3$H]-VCR exposure.

To terminate accumulation of [$^3$H]-VCR, the cells were aspirated dry, washed with PBS at 4°C, and lysed with 2 ml of 1 N NaOH. Lysis was carried out for 12 h at 37°C then 1.6 ml of lysate was transferred to a scintillation vial, mixed with 10 ml scintillation fluid (Ultima Gold, Packard), and counted for tritium in a liquid scintillation counter (2200CA, Packard). The remaining 0.4 ml was used to determine protein concentration using the Lowry method (Lowry et al, 1951). From these measurements the accumulation of [$^3$H]-VCR per mg protein was calculated.

### RESULTS

### Characterization

Expression of three genes implicated in multidrug resistance (*mdr-1*, *mrp-1* and *lrp*) was assayed at both the protein and mRNA level in the rhabdomyosarcoma cell line panel. Expression of P-gp and MRP proteins was not detectable in the rhabdomyosarcoma cell lines by immunoblotting (Figure 1) whereas the control cell lines gave a strong signal.

Immunohistochemical staining showed that expression of MDR proteins was undetectable or very low in all of the cell lines except HX170 which has expression of LRP (Table 2A). In many cases the staining was barely detectable, as illustrated in Figure 2. Despite the difficulties in detecting proteins, many of the cell lines were found to contain mRNA for the resistance genes (Table 2B). *mdr-1*, *mrp-1* and *lrp* were found in 5/7, 7/7 and 5/7 cell lines, respectively. The detection of these is probably due to the high sensitivity of the PCR assay compared to the protein detection methods.

The *p53* phenotype of the cell line panel was investigated by measuring levels of P53, P21 and MDM2 4 h following 5 Gy irradiation and comparing these to untreated controls (Figure 3). The α-tubulin levels vary between cell lines but are consistent within cell-line paired samples, showing that loading was reasonably accurate. The experiment was repeated twice and the blots analysed using ImageQuant software.

In the wild-type control, A2780, there is an increase in expression of MDM2 and P53 leading to a downstream increase in P21, consistent with the presence of functional *p53*. Of the rhabdomyosarcoma cell lines only one, SCMC, appears to be functionally wild-type for *p53*. In HX170 an increase in P21 is seen following irradiation but there is no corresponding increase in P53 so it is unclear whether *p53* is functional. An increase in P21 levels following irradiation is also seen in RMS, possibly indicating some *p53* function. Rh18 and Rh36 showed overexpression of MDM2 leading to high levels of P53 and P21 and no response following irradiation. Both Rh30 and RD had high levels of P53 prior to irradiation and showed no induction of P21, suggesting mutant *p53*.

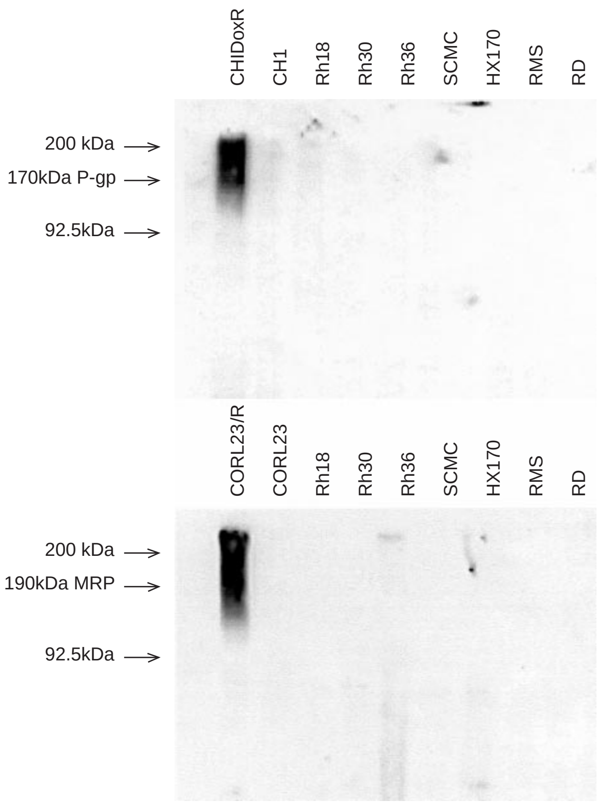

**Figure 1** Typical immunoblots for P-gp and MRP in a panel of human rhabdomyosarcoma cell lines and control cell lines

**Table 2** (**A**) Expression of MDR-related proteins in rhabdomyosarcoma lines by immunohistochemistry. (**B**) Expression of MDR-related genes in rhabdomyosarcoma lines by RT-PCR. – negative, + positive, –/+ unclear

| | P-gp | MRP | LRP |
|---|---|---|---|
| **A** | | | |
| Rh18 | – | –/+ | –/+ |
| Rh30 | – | – | – |
| Rh36 | – | –/+ | –/+ |
| SCMC | –/+ | – | – |
| HX170 | –/+ | – | + |
| RMS | – | – | – |
| RD | – | –/+ | –/+ |
| **B** | **P-gp** | **MRP** | **LRP** |
| Rh18 | – | + | + |
| Rh30 | + | + | + |
| Rh36 | + | + | + |
| SCMC | + | + | – |
| HX170 | + | + | + |
| RMS | – | + | – |
| RD | + | + | + |

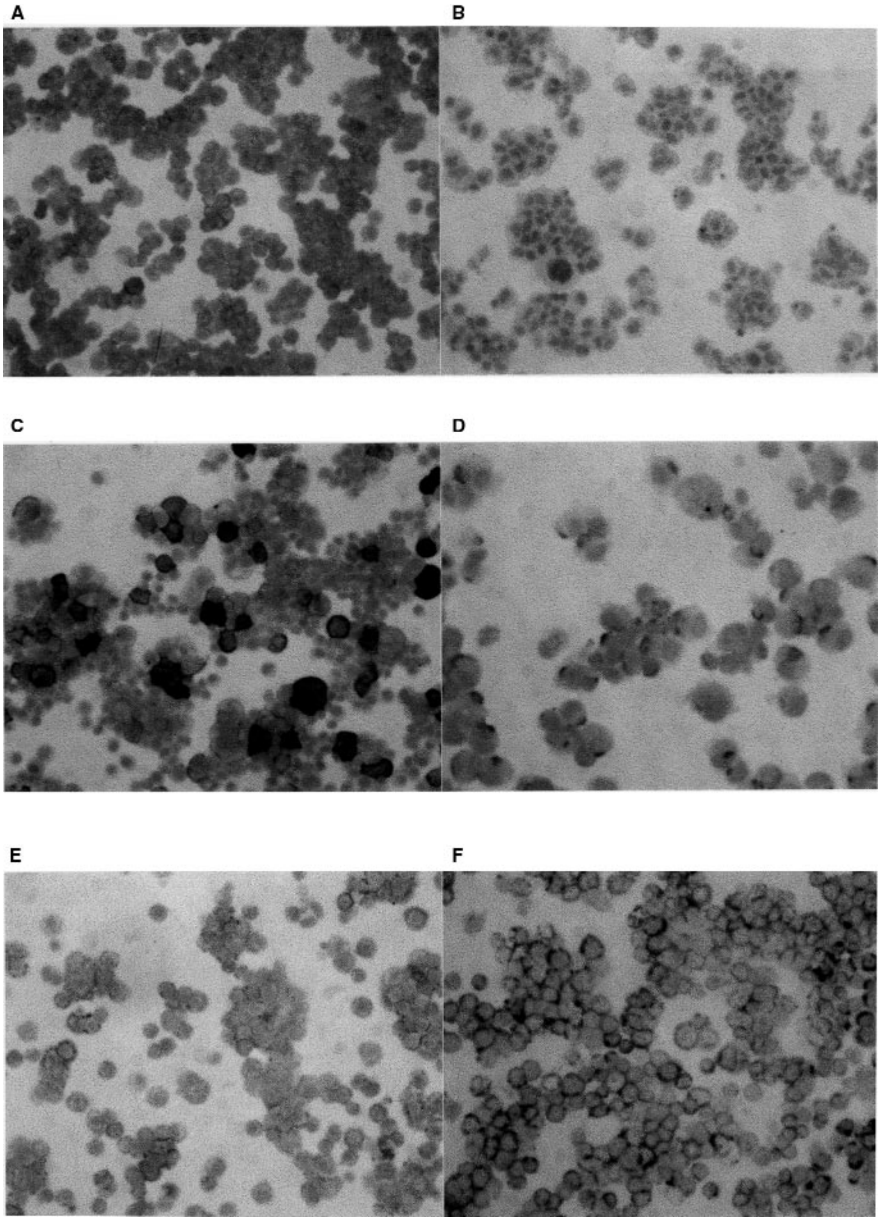

**Figure 2** Example immunohistochemistry results. (**A**) CH1DoxR and (**B**) RD stained for P-gp using MRK 16, (**C**) CORL23R and (**D**) Rh36 stained for MRP using MRPm6; (**E**) 2R120 and (**F**) HX170 stained for LRP using LRP56

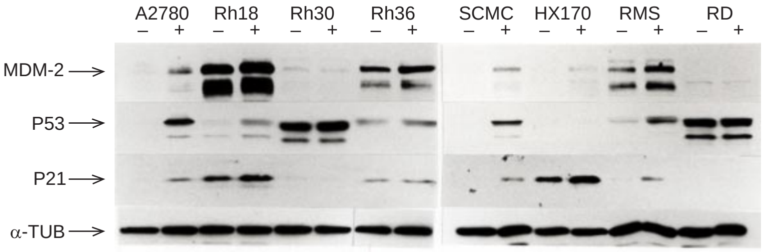

**Figure 3** Typical immunoblot showing levels of MDM2, P53, P21 and α-tubulin in the rhabdomyosarcoma cell lines and A2780 control. – untreated; + 4 h following 5 Gy irradiation

 *© 2000 Cancer Research Campaign*

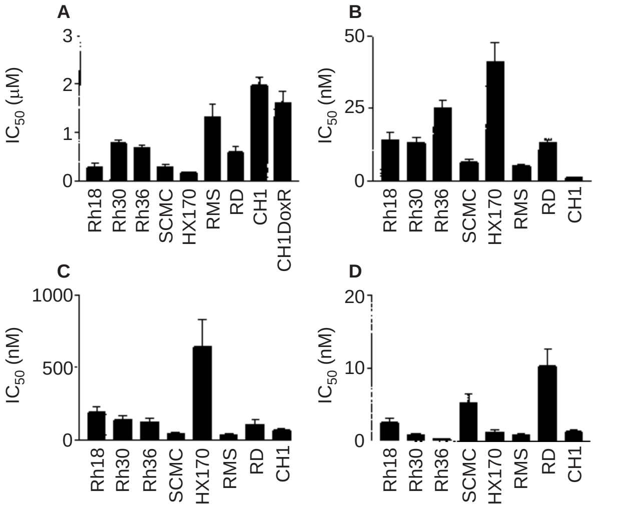

**Figure 4** Sensitivity of a panel of human rhabdomyosarcoma cell lines to: (**A**) cisplatin, (**B**) doxorubicin, (**C**) etoposide and (**D**) vincristine. $IC_{50}$ values given are the mean ± standard deviation from three independent experiments. CH1DoxR $IC_{50}$ values not shown; doxorubicin (414±42 nM), etoposide (2390±710 nM) and vincristine (291±32 nM)

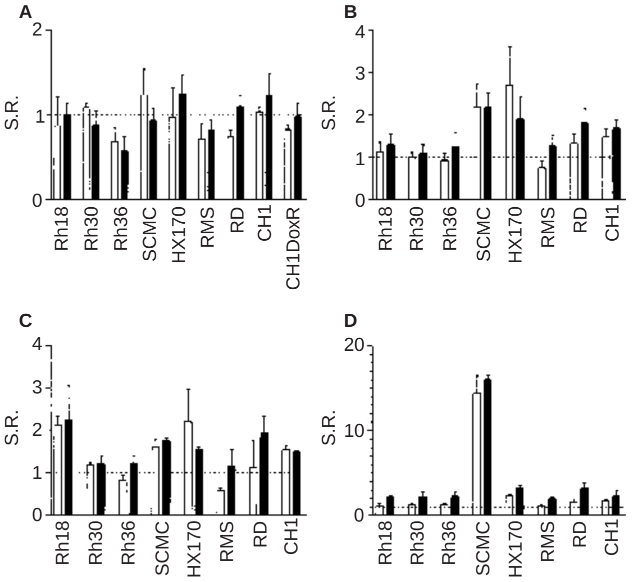

**Figure 5** Modulation of sensitivity of a panel of rhabdomyosarcoma cell lines to: (**A**) cisplatin, (**B**) doxorubicin, (**C**) etoposide and (**D**) vincristine using 2 μM PSC833 (open bars) or VX710 (filled bars). Values given are the mean ± standard deviation of three independent experiments. Sensitization ratio (SR) = $IC_{50}$ in the absence of modulator/$IC_{50}$ in the presence of modulator. Dotted line represents SR = 1 (no sensitization). Values not shown for CH1DoxR; SR with PSC833 and doxorubicin (53.9 ± 5.5), etoposide (17.8 ± 2.4), vincristine (455 ± 51); SR with VX710 and doxorubicin (12.3 ± 2.0), etoposide (1.85 ± 0.07), vincristine (13.4 ± 2.5)

### Sensitivity to anti-cancer agents

A growth inhibition assay was used to measure the sensitivity of the rhabdomyosarcoma cell lines to the commonly used agents cisplatin, doxorubicin, etoposide and vincristine (Figure 4A–D, respectively). For cisplatin, the $IC_{50}$ values range from 0.2–2 μM and are similar for all the lines including CH1DoxR which over-expresses P-gp. For doxorubicin, etoposide and vincristine there is greater variability in the $IC_{50}$ values although the rhabdomyosarcoma cell lines are considerably more sensitive than CH1DoxR. In the rhabdomyosarcoma lines the $IC_{50}$ values range from 5.4–41.2 nM for doxorubicin, 38–650 nM for etoposide and 0.3–10.3 nM for vincristine.

Overall, there was no clear correlation between drug sensitivity and *p53*/*mdm2* status, although SCMC, which has wild-type *p53* and is derived from a heavily treated patient, was relatively sensitive to the DNA-damaging agents cisplatin, etoposide and doxorubicin. The line HX170 exhibits an interesting pattern of drug sensitivity as it is most resistant to doxorubicin and etoposide but relatively sensitive to cisplatin and vincristine.

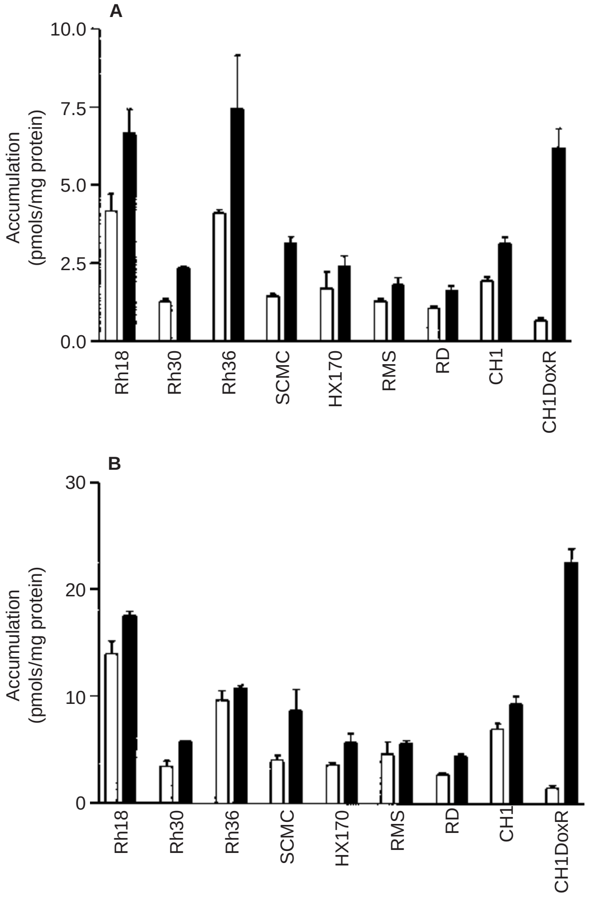

**Figure 6** Accumulation of [$^3$H]-VCR by a panel of rhabdomyosarcoma cell lines incubated with: (**A**) 10 nM [$^3$H]-VCR, (**B**) 25 nM [$^3$H]-VCR in the absence of modulator (open bars) or with 2 μM PSC833 (closed bars). Values are mean ± standard deviation from three experiments

### Modulation of sensitivity

The ability of PSC833 and VX-710 to modulate sensitivity to cisplatin, doxorubicin, etoposide and vincristine was measured (Figure 5). A sensitization ratio greater than 1 indicates that modulation is taking place. For cisplatin the sensitization ratios are all approximately 1 (Figure 5A). This would be expected because cisplatin is not a substrate for P-gp or MRP. With etoposide (Figure 5C) some sensitization is seen in HX170, SCMC, RD and Rh18 but this is low (approximately 2-fold) compared to the sensitization in CH1DoxR (up to 17.8-fold). The amount of sensitization seen in CH1DoxR is greatest with doxorubicin and vincristine

© 2000 Cancer Research Campaign

(Figure 5B and 5D) and is as much as 455-fold. The sensitization seen in the rhabdomyosarcoma lines is considerably less than this but is as high as 16-fold in SCMC with vincristine. Sensitization is also seen at lower levels in HX170 and RD.

The modulator PSC833 is more potent in modulation of VCR sensitivity in the CH1DoxR ovarian line whereas the modulator VX710 is significantly more potent in the rhabdomyosarcoma lines (paired *t*-test, $P < 0.0001$).

The sensitization by the modulators is greatest with vincristine and this may be due to the uptake and efflux mechanisms for this drug. To investigate this, accumulation of 10 nM and 25 nM [$^3$H]-VCR was measured both in the presence and absence of PSC833. PSC833 caused an increase in accumulation in all rhabdomyosarcoma lines from 1.1-fold in Rh36 to 2.2-fold in SCMC (Figure 6). Interestingly, the greatest increase was seen in SCMC, but this was not statistically significant. The accumulation of [$^3$H]-VCR by CH1DoxR was lower than all the other lines but could be increased up to 15-fold by addition of PSC833.

### DISCUSSION

Rhabdomyosarcoma is an important childhood malignancy where drug resistance to the major drugs used to treat the disease (vincristine, etoposide and doxorubicin) limits cure rates. This study reports the characterization of a panel of human cell lines in terms of the major proteins which may influence drug response, chemosensitivity and sensitization by two clinically studied modulators of multidrug resistance.

The rhabdomyosarcoma cell line panel comprises lines derived from both previously untreated and chemotherapy-treated patients and displays a wide variety of phenotypes. There are similar numbers of lines of alveolar and embryonal histology. All of the lines tested negative for P-gp and MRP by immunoblotting although it is likely that this technique is not sensitive enough to identify low levels of the proteins. This is supported by the results of immunohistochemical staining, which suggests that the level of proteins is very low and barely detectable, with the exception of HX170 which expresses LRP.

Consequently, RT-PCR has also been used and shows that the majority of lines have *mdr-1*, *mrp-1* and *lrp* mRNA. Similar observations have also been made in neuroblastoma (Yanagisawa et al, 1999). However, the expression of mRNA does not always reflect the expression of the protein.

Our studies of the *p53* pathway are based on the model that wild-type *p53* induces P21 expression following irradiation and that therefore induction of P21 is indicative of wild-type *p53* (O’Connor et al 1997). However, it should be noted that alternate mechanisms may also be involved and that our observations therefore only provide a suggestion of *p53* status.

Clinical observations suggest that the function of *p53* is often compromised in paediatric rhabdomyosarcoma (Wurl et al, 1996). In agreement with this, it is evident from our studies that only one of the seven rhabdomyosarcoma lines, SCMC, shows P21 induction following irradiation, suggesting functional *p53*. Interestingly, this cell line was relatively sensitive to the DNA-damaging agents cisplatin, etoposide and doxorubicin, and this is in agreement with data using the 60 cell-line NCI panel to correlate *p53* status and drug sensitivity (O’Connor et al, 1997). HX170 also shows some induction of P21 but its status is unclear. Rh30 and RD have mutations in exons 8 and 7 respectively (Felix et al, 1992; Keleti et al, 1996). Our results suggest that these mutations lead to a loss of *p53* function, as illustrated by the lack of P21 induction following irradiation. Two of the lines, Rh18 and Rh36, show overexpression of MDM2, confirming previously published data (Felix et al, 1992; Keleti et al, 1996). We hypothesize that the overexpression of MDM2 leads to inactivation of P53 and thus no induction of P21 following irradiation. Previously published reports have shown that RMS has a point mutation in the *p53* gene (Stratton et al, 1990). We have observed a slight induction of P21 following irradiation in this line. We therefore propose that this mutation may not disable *p53* function completely or may be heterozygous.

Neither the presence of MDR proteins nor *p53* status of these lines appeared to correlate with sensitivity to commonly used chemotherapeutic agents. HX170 showed reduced sensitivity to cisplatin, doxorubicin and etoposide but not to vincristine, and SCMC and RD were relatively resistant to vincristine alone. Interestingly, all three lines are derived from heavily treated patients and all three lines show some modulation of sensitivity. Only two of the lines, Rh18 and Rh30, were taken from untreated patients and neither of these showed any modulation. Four of the lines from treated patients showed modulation, as high as 16-fold in SCMC. The level of modulation seen in these lines is relatively low and therefore it is likely that the MDR proteins present are at low levels. This may also be more relevant to clinical levels of expression, where levels of modulation are also low (Cowie et al, 1995).

Sensitization of the rhabdomyosarcoma lines to vincristine was greater with VX710 than PSC833, in contrast to the P-gp-expressing ovarian line CH1DoxR. This suggests that the rhabdomyosarcoma lines may also have low levels of MRP which is affected by VX710 but to a lesser effect by PSC833 (Germann et al, 1997*b*). This is supported by the observation that all the cell lines express mrp1 mRNA (Table 2).

The ability of the cells to accumulate radiolabelled vincristine was measured both in the absence and presence of PSC833. In all cell lines PSC833 increased accumulation of vincristine, regardless of MDR phenotype. This implies that there may be low levels of transport proteins in all the cells which are affected by PSC833. Alternatively, PSC833 may act via a different mechanism to increase drug accumulation. One hypothesis is that exposure to drugs activates a stress response pathway which activates P-gp and/or *mdr-1* production (Chaudhary and Roninson, 1993). Therefore the MDR phenotype of the unstressed cell does not reflect the situation when drugs are added and PSC833 may interact with the stress response pathway.

In conclusion, the rhabdomyosarcoma cell lines studied do not show high levels of multidrug resistance. Despite the lack of easily detectable MDR proteins, there is evidence that the proteins are present at very low levels. There is also evidence that the sensitivity of the rhabdomyosarcoma lines to drugs, especially vincristine, can be modulated, by as much as 16-fold in SCMC, and that accumulation of vincristine can also be increased using modulators. This may have clinical implications in terms of excluding P-gp and MRP ‘non-expressors’ from clinical trials of modulating agents or looking for clear correlations between expression and modulation benefit. These lines provide a good model for the development of new strategies in the treatment of paediatric rhabdomyosarcoma and for elucidating relationships between protein expression and drug resistance.

© 2000 Cancer Research Campaign

### ACKNOWLEDGMENTS

This work is supported by an Institute of Cancer Research studentship. We thank Dr K Pritchard-Jones for advice and discussion and A Newman, J Renshaw and S Sharp for technical guidance. We thank Vertex pharmaceuticals and Sandoz Pharmaceuticals for donating VX-710 and PSC833 respectively.

### REFERENCES

Barrand MA, Heppell-Parton AC, Wright KA, Rabbitts PH and Twentyman PR (1994) A 190-kDa protein overexpressed in non-P-glycoprotein-containing multidrug-resistant resistant cells and its relationship to the MRP gene. *J Natl Cancer Inst* **86**: 110–117

Bordow SB, Haber M, Madafiglio J, Cheung B, Marshall GM and Norris MD (1994) Expression of the multidrug resistance-associated protein (MRP) gene correlates with amplification and overexpression of the N-myc oncogene in childhood neuroblastoma. *Cancer Res* **54**: 5036–5040

Chan HSL, Thorner PS, Haddad G and Ling V (1990) Immunohistochemical detection of P-glycoprotein – Prognostic correlation in soft-tissue sarcoma of childhood. *J Clin Oncol* **8**: 689–704

Chaudhary and Roninson (1993) Induction of multidrug resistance in human cells by transient exposure to different chemotherapeutic drugs. *J Natl Cancer Inst* **85**: 632–639

Cole SPC, Bhardwaj G, Gerlach JH, Mackie JE, Grant CE, Almquist KC, Stewart AJ, Kurz EU, Duncan AMV and Deeley RG (1992) Overexpression of a transporter gene in a multidrug-resistant human lung cancer cell line. *Science* **258**: 1650–1654

Cowie FJ, Pinkerton CR, Phillips M, Dick G, Judson I, McCarthy PT and Flanagan RJ (1995) Continuous-infusion verapamil with etoposide in relapsed or resistant paediatric cancers. *Br J Cancer* **71**: 877–881

Cowie FJ, Pritchard-Jones K, Renshaw J and Pinkerton CR (1998) Multidrug resistance modulation in rhabdomyosarcoma and neuroblastoma cell lines. *Int J Oncol* **12**: 1143–1149

Douglass EC, Valentine M, Etcubanas E, Parham D, Webber BL, Houghton PJ, Green AA and Houghton JA (1987) A specific chromosomal abnormality in rhabdomyosarcoma. *Cytogenet Cell Genet* **45**: 148–155

Enzinger FM and Weiss SW (1983) *Soft Tissue Tumours*. CV Mosby Co: St. Louis

Enzinger FM and Weiss SW (1995) *Soft Tissue Tumours*. 3rd Edn. CV Mosby Co: St. Louis

Felix CA, Chavez Kappel C, Mitsudomi T, Nau MM, Tsokos M, Crouch GD, Nisen PD, Winick NJ and Helman LJ (1992) Frequency and diversity of p53 mutations in childhood rhabdomyosarcoma. *Cancer Res* **52**: 2243–2247

Ford JM (1996) Experimental reversal of P-glycoprotein-mediated multidrug resistance by pharmacological chemosensitisers. *Eur J Cancer* **32A**: 991–1001

Garvin AJ, Stanley WS, Bennett DD, Sullivan JL and Sens DA (1986) The *in vitro* growth, heterotransplantation, and differentiation of a human rhabdomyosarcoma cell line. *Am J Pathol* **125**: 208–217

Germann UA, Shlyakhter D, Mason VS, Zelle RE, Duffy JP, Galullo V, Armistead DM, Saunders JO, Boger J and Harding MW (1997*a*) Cellular and biochemical characterization of VX-710 as a chemosensitizer: reversal of P-glycoprotein-mediated multidrug resistance *in vitro*. *Anticancer Drugs* **8**: 125–140

Germann UA, Ford PJ, Shlyakhter D, Mason VS and Harding MW (1997*b*) Chemosensitization and drug accumulation effects of VX-710, verapamil, cyclosporin A, MS-209 and GF120918 in multidrug resistant HL60/ADR cells expressing the multidrug resistance-associated protein MRP. *Anticancer Drugs* **8**: 141–155

Hayashi Y, Sugimoto T, Horii Y, Hosoi H, Inazawa J, Kemshead JT, Inaba T, Hanada R, Yamamoto K, Gown AM and Sawada T (1990) Characterization of an embryonal rhabdomyosarcoma cell line showing amplification and over-expression of the N-*myc* oncogene. *Int. J. Cancer* **45**: 705–711

Houghton JA, Houghton PJ and Webber BL (1982) Growth and characterization of childhood rhabdomyosarcoma as xenografts. *J Natl Cancer Inst* **68**: 437–443

Keleti J, Quezado MM, Abaza MM, Raffeld M and Tsokos M (1996) The MDM2 oncoprotein is overexpressed in rhabdomyosarcoma cell lines and stabilizes wild-type p53 protein. *Am J Pathol* **149**: 143–151

Kelland LR, Bingle L, Edwards S and Steel GG (1989) High intrinsic radiosensitivity of a newly established and characterised human embryonal rhabdomyosarcoma cell line. *Br J Cancer* **59**: 160–164

Kondo S, Kondo Y, Hara H, Kaakaji R, Peterson JW, Morimura T, Takeuchi J and Barnett GH (1996) *mdm2* gene mediates the expression of *mdr1* gene and P-glycoprotein in a human glioblastoma cell line. *Br J Cancer* **74**: 1263–1268

Kuttesch JF, Parham DM, Luo X, Meyer WH, Bowman L, Shapiro DN, Pappo AS, Crist WM, Beck WT and Houghton PJ (1996) P-Glycoprotein expression at diagnosis may not be a primary mechanism of therapeutic failure in childhood rhabdomyosarcoma. *J Clin Oncol* **14**: 886–900

Lane DP (1992) p53, Guardian of the genome. *Nature* **358**: 15–16

Lowry OH, Rosebrough NJ, Lewis Farr A and Randall RJ (1951) Protein measurements with the folin phenol reagent. *J Biol Chem* **193**: 265–275

McAllister RM, Melnyk J, Finkelstein JZ, Adams EC and Gardner MB (1969) Cultivation in vitro of cells derived from a human rhabdomyosarcoma. *Cancer* **24**: 520–526

Mosmann T (1983) Rapid colorimetric assay for cellular growth and survival: application to proliferation and cytotoxicity assays. *J Immunol Methods* **65**: 55–63

Naito M and Tsuruo T (1997) New multidrug-resistance-reversing drugs, MS-209 and SDZ PSC833. *Cancer Chemother Pharmacol* **40**: S20–S24

O'Connor PM, Jackman J, Bae I, Myers TG, Fan S, Mutoh M, Scudiero DA, Monks A, Sausville EA, Weinstein JN, Friend S, Fornace AJ and Kohn KW (1997) Characterization of the *p53* tumour suppressor pathway in cell lines of the National Cancer Institute anticancer drug screen and correlations with the growth-inhibitory potency of 123 anticancer agents. *Cancer Res* **57**: 4285–4300

Pappo AS, Shapiro DN, Crist WM and Maurer HM (1995) Biology and therapy of paediatric rhabdomyosarcoma. *J Clin Oncol* **13**: 2123–2139

Pestell KE, Medlow CJ, Titley JC, Kelland LR and Walton MI (1998) Characterisation of the *P53* status, BCL-2 expression and radiation and platinum drug sensitivity of a panel of human ovarian cancer cell lines. *Int J Cancer* **77**: 913–918

Pinkerton CR (1996) Multidrug resistance reversal in childhood malignancies – potential for a real step forward? *Eur J Cancer* **32A**: 641–644

Scheper RJ, Broxterman HJ, Scheffer GL, Kaaijk P, Dalton WS, van Heijningen THM, van Kalken CK, Slovak ML, de Vries EGE, van der Valk P, Meijer CJLM and Pinedo HM (1993) Overexpression of a Mr 110,000 vesicular protein in non-P-glycoprotein-mediated multidrug resistance. *Cancer Res* **53**: 1475–1479

Sharp SY, Rowlands MG, Jarman M, and Kelland LR (1994) Effects of a new antioestrogen, idoxifene, on cisplatin- and doxorubicin-sensitive and -resistant human ovarian carcinoma cell lines. *Br J Cancer* **70**: 409–414

Sharp SY, Rogers P and Kelland LR (1995) Transport of cisplatin and bis-acetato-ammine dichlorocyclohexamine platinum (IV) (JM216) in human ovarian carcinoma cell lines: Identification of a plasma membrane protein associated with cisplatin resistance. *Clin Cancer Res* **1**: 981

Stein U, Walther W, Laurencot CM, Scheffer GL, Scheper RL and Shoemaker RH (1997) Tumour necrosis factor-a and expression of the multidrug resistance associated genes LRP and MRP. *J Natl Cancer Inst* **89**: 807–813

Stratton MR, Moss S, Warren W, Patterson H, Clark J, Fisher C, Fletcher CDM, Ball A, Thomas M, Gusterson BA and Cooper CS (1990) Mutation of the p53 gene in human soft tissue sarcomas: association with abnormalities of the RB1 gene. *Oncogene* **5**: 1297–1301

Towbin H, Staehelin T and Gordon J (1979) Electrophoretic transfer of proteins from polyacrylamide gels to nitrocellulose sheets: procedures and some applications. *Proc. Natl Acad Sci USA* **76**: 350

Wurl P, Taubert H, Bache M, Kroll J, Meye A, Berger D, Siermann A, Holzhausen H-J, Hinze R, Schmidt H and Rath F-W (1996) Frequent occurrence of p53 mutations in rhabdomyosarcoma and leiomyosarcoma, but not in fibrosarcoma and malignant neural tumours. *Int J Cancer* **69**: 317–323

Yanagisawa T, Newman A, Coley H, Renshaw J, Pinkerton CR and Pritchard-Jones K (1999) Biricodar (VX-710; Incel (TM)): An effective chemosensitiser in neuroblastoma. *Br J Cancer* **80**: 1190–1196

© 2000 Cancer Research Campaign
